# The Portuguese experience on regionalisation of perinatal care

**DOI:** 10.1186/1824-7288-40-S2-A8

**Published:** 2014-10-09

**Authors:** Hercilia Guimarães

**Affiliations:** 1 Faculty of Medicine of Porto University, Serviço de Neonatologia, Hospital de São João, Porto, Portugal

## 

In order to improve perinatal outcomes, national guidelines proposed by the National Committee for Mother and Child Health, were promulgated by the Ministry of Health in 1990. A Child and Maternal Hospital Healthecare Referral Network was created. This document advised for some innovating and frontal aspects: 1. Maternities with less than 1500 deliveries per year should be closed; 2. Hospitals were classified as Perinatal Care Hospitals (Level II- able to provide care to pregnant women and normal newborns, and should include a Neonatal Internediate Care Unit) and Differentiated Perinatal Care Hospitals (Level III-to provide care to high risk pregnant womenand newborn infants, and should include a Neonatal Intensive Care Unit); 3. Functional Coordinang Units were created to connect the Hospitals to the Primary Healcare Centres; 4. Cycles of Special Studies on Neonatology were created to graduate Paediatricians in Neonatology; 5. The recognition that the best transport for the newborn is the mother´s womb; however, in 1987, a Neonatal Transport was created to unavailable situations.

Both pregnant women and newborns are transferred according to the following priority: pathology, geographical referral and available vacancy.

Another importante aspect was the organization of paediatricians and neonatologists (1985) as scientific societies and the publication of national protocols as an attempt to standardize methodologies.

The National Registry of VLBWinfants, inspired on Vermont-Oxford Network, was an initiative started in 1994 with a voluntary participation of the NICU´s.

All these aspects had clinical implications, namely in decreasing mortality rates, as shown in table 1.

**Table 1:** 

Mortalities	1980	1990	2000	2010	2012
Maternal /100000	19.0	10.3	2.5	7.9	4.5
Late Foetal	11.8	8.6	3.7	2.4	2.8
Neonatal	15.5	7.0	3.4	1.7	2.2
Early neonatal	12.3	4.7	2.5	1.1	1.5
Infant	24.3	10.9	5.5	2.5	2.9 (2013)

In conclusion, the reform of perinatal care in Portugal is an exemple of how a good diagnosis and adequate proposals combined with a strong political will are crucial for changing.

